# Mixture prior distributions and Bayesian models for robust radionuclide image processing

**DOI:** 10.3389/fnume.2024.1380518

**Published:** 2024-09-05

**Authors:** Muyang Zhang, Robert G. Aykroyd, Charalampos Tsoumpas

**Affiliations:** ^1^ Department of Statistics, School of Mathematics, University of Leeds, Leeds, United Kingdom; ^2^ Department of Nuclear Medicine and Molecular Imaging, University Medical Center Groningen, University of Groningen, Groningen, Netherlands

**Keywords:** medical imaging, Bayesian methods, machine learning, inhomogeneous models, Markov chain Monte Carlo

## Abstract

The diagnosis of medical conditions and subsequent treatment often involves radionuclide imaging techniques. To refine localisation accuracy and improve diagnostic confidence, compared with the use of a single scanning technique, a combination of two (or more) techniques can be used but with a higher risk of misalignment. For this to be reliable and accurate, recorded data undergo processing to suppress noise and enhance resolution. A step in image processing techniques for such inverse problems is the inclusion of smoothing. Standard approaches, however, are usually limited to applying identical models globally. In this study, we propose a novel Laplace and Gaussian mixture prior distribution that incorporates different smoothing strategies with the automatic model-based estimation of mixture component weightings creating a locally adaptive model. A fully Bayesian approach is presented using multi-level hierarchical modelling and Markov chain Monte Carlo (MCMC) estimation methods to sample from the posterior distribution and hence perform estimation. The proposed methods are assessed using simulated $\gamma {\text{-eye}}^{\text{TM}}$ camera images and demonstrate greater noise reduction than existing methods but without compromising resolution. As well as image estimates, the MCMC methods also provide posterior variance estimates and hence uncertainty quantification takes into consideration any potential sources of variability. The use of mixture prior models, part Laplace random field and part Gaussian random field, within a Bayesian modelling approach is not limited to medical imaging applications but provides a more general framework for analysing other spatial inverse problems. Locally adaptive prior distributions provide a more realistic model, which leads to robust results and hence more reliable decision-making, especially in nuclear medicine. They can become a standard part of the toolkit of everyone working in image processing applications.

## 1 Introduction

Radionuclide imaging is widely used for the diagnosis of several diseases and monitoring their treatment (1–4). However, the images inherently suffer from relatively limited resolution due to motion, collimator size, and scatter, as well as noise due to limited statistics (5, 6). The true biological image can be approximated by incorporating a transformation matrix. However, this matrix is too large and ill-posed to obtain the exact image by directly solving a system of linear equations.

Image processing methods are commonly used to solve such ill-posed inverse problems. In medical imaging, one can derive a new image of the unknown emitter activity X from the measured data P by solving the inverse problem. The measured data are related to “the actual activity” with form E[P]=f(X) being identified as suitable for many different image applications (7, 8). However, depending on the transformation we are looking into, f(X) can turn to a linear function with a projection matrix or known non-linear transformation function, especially when time and multi-layers factor, including spread functions (9–11), Kernel functions (12, 13), and wavelet and Fourier functions (9, 14). The aim of this project was to improve image quality, having obtained prior information regarding the underlying image (e.g., the method of image acquisition and the type of noise). We propose making use of posterior distributions with knowledge-based prior distributions, which are designed under a Bayesian framework. Our approach will be demonstrated in synthetic data derived from actual acquired radionuclide imaging data.

## 2 Methods of image processing

### 2.1 Bayesian modelling

We consider solving the linear inverse problem of calculating X from Y, having m and n elements, respectively, with transformation matrix A, of size n×m, consisting of elements $a_{ij}$. These are related by

(1) E[Y]=AX,

with image data noise, for example, Gaussian or Poisson, depending on the type of scanning system being used.

#### 2.1.1 Likelihood function

Assuming the image data from $\gamma {\text{-eye}}^{\text{TM}}$ follows a Poisson distribution, then the conditional distribution for observation Y given the unknown true image X is given by

(2) $$f_{Y|X}\left(y_{1},y_{2},\ldots .y_{n}|x\right)=\prod_{\;i=1}^{n}\lambda_{i}^{y_{i}}\frac{\exp\left(-\lambda_{i}\right)}{Y_{i}!},$$

where $E[Y_{i}]=\lambda_{i}=\sum_{\;j=1}^{m}a_{ij}x_{j}$, j=1,2,…,m. In other words, each projection data value $y_{i}$ has an according interaction with the whole vector X (8).

#### 2.1.2 Prior distribution

The prior distribution in our Bayesian application for image processing follows the Gibbs form defining a Markov random field (MRF). The variables in an MRF are only related to their adjacent neighbours while being conditionally independent of the others (15).The corresponding prior density is given by

(3) $$\pi_{X}\left(x|B\right)=Z^{-1}\exp\left(-B\kappa \left(x\right)\right),\;Z=\int_{x}\exp\left(-B\kappa \left(x\right)\right)dx,\;X\in R^{m},B>0,$$

where Z is the normalisation for the Gibbs distribution; the energy function is κ (15), representing the energy of the configuration of pixels, and B is a non-negative smoothing parameter (16–18). Furthermore, the energy function can be rewritten as the sum of local energy functions Φ(⋅):

(4) $$\kappa \left(x\right)=\sum_{\;j=1}^{m}\Phi_{j}\left(x\right),$$

where $\Phi_{\;j}(\cdot )$ represents the local energy function corresponding to $X_{j}=x_{j}$. Here, the first order of an MRF, which consists of four closest neighbours (up, down, left, and right), is taken into consideration.The linear combination of the candidate and its closest neighbours is denoted as

(5) $$\Phi_{\;j}\left(x\right)=\sum_{t\in \partial \left(\;j\right)}w_{\;jt}\phi \left(x_{j}-x_{t}\right),$$

where $w_{\;jt}=0.5$ in the first-order MRF. The set of nodes ∂(j) forms a finite graph X with edges j∼t (15). Finally, after employing an MRF for pixel difference, the prior distribution is now written as

(6) $$\pi_{X}\left(x|B\right)=Z^{-1}\exp\left(-B\sum_{\;j=1}^{m}\sum_{t\in N_{\partial \left(\;j\right)}}\;w_{\;jt}\phi \left(x_{j}-x_{t}\right)\right).$$

Mathematical forms within the potential functions can assign priors with different properties. For instance, the two most common cases are the absolute value and quadratic functions: ϕ(μ)=|μ| and $\phi (\mu )=\mu^{2}$, respectively. Thereby, the corresponding priors are an MRF with an absolute function [corresponding to a Laplace MRF (LMRF)] and a quadratic potential function [corresponding to a Gaussian MRF (GMRF)], respectively:

(7) $$\pi_{X|\tau }\left(x|\tau \right)=\left\{ \begin{array}{cc} \frac{1}{{\left(2\tau \right)}^{m}}\exp\left(-\frac{\sum_{\;j=1}^{m}\sum_{t\in \partial \left(\;j\right)}\;|x_{j}-x_{t}|}{\tau }\right),\; & x_{j}\geq 0,\tau >0;\;\\ \frac{1}{{\left(\sqrt{2\pi }\tau \right)}^{m}}\exp\left(-\frac{\sum_{\;j=1}^{m}\sum_{t\in \partial \left(\;j\right)}\;{\left(x_{j}-x_{t}\right)}^{2}}{2\tau^{2}}\right),\; & x_{j}\geq 0,\tau >0.\; \end{array}\right.$$

This representation assumes that there is a high similarity between a pixel and its neighbouring pixels. The prior mean is expected to be zero, and the prior conditional variance is τ=1/B. Here, as τ is a global prior variance parameter, the potential function including τ also retains the consistent principal for an MRF: $\phi (x_{j}-x_{t})=\phi (x_{t}-x_{j})$. The constant terms are $k=1/2^{m}$ and $k=1/{\left(2\pi \right)}^{m/2}$ in each case, respectively.

#### 2.1.3 Introduction of hyperprior distribution

It is common to introduce an uninformative or weakly informative prior for prior parameters, such as τ, like the uniform distribution and other flat priors, especially when there is a lack of information in advance. Nonetheless, a flat prior permits outcomes with equal possibilities; this type of prior may lead to a posterior distribution with many equally likely outcomes that is an improper distribution, and the estimation would fail to realise convergence.

As there is no supportive information about these variances beforehand, we introduce a weakly informative hyperprior distribution π(τ)∝1/τ. This type of prior includes a Jacobian transformation that was first suggested by DeGroot and Lindstrom (19). It has since been widely used in many non-informative cases. In general, the idea is to assign a uniform prior with an even probability p to a logarithmic transformation of the unknown non-negative parameter, represented as t=log(τ) and f(t)∝p. Thereby, the probability for τ is proportional to the Jacobian transformation (dt/dτ) from t to τ: π(τ)∝p⋅(dt/dτ)∝1/τ. The completed posterior description, after employing a hyperprior of Laplace and Gaussian types, respectively, is given by

(8) $$\begin{array}{rl} \;f_{X,\tau |Y}\left(x,\tau |y\right) & \propto f_{Y|X}\left(y|x\right)\;\pi_{X|\tau }\left(x|\tau \right)\pi \left(\tau \right)\\ & \;\propto \left\{ \begin{array}{c} \prod_{i=1}^{n}\frac{{\left(\sum_{i}a_{i\;j}\;x_{j}\right)}^{y_{i}}}{\tau^{d}}\exp\left(-\sum_{\;j=1}^{m}\left(\sum_{i}a_{i\;j}\;x_{j}\right)-\frac{\sum_{t\in N_{\partial \left(\;j\right)}}\;|x_{j}-x_{t}|}{\tau }\right)\frac{1}{\tau };\;\\ \prod_{i=1}^{n}\frac{{\left(\sum_{i}a_{i\;j}\;x_{j}\right)}^{y_{i}}}{\tau^{d}}\exp\left(-\sum_{\;j=1}^{m}\left(\sum_{i}a_{i\;j}\;x_{j}\right)-\frac{\sum_{t\in N_{\partial \left(\;j\right)}}\;{\left(x_{j}-x_{t}\right)}^{2}}{2\tau^{2}}\right)\frac{1}{\tau }.\; \end{array}\right. \end{array}$$

When estimating the parameter τ, we can regard X as a known parameter and then update τ from $f_{X,\tau }(x,\tau )\propto \pi (x|\tau )\pi (\tau )$ by MCMC estimation. Hence, we can divide the inference of full joint distribution π(x,τ∣y) into two successive steps. Multivariate has a broader application in comparison with univariate distribution in reality. The MCMC sampling method can be extended to the hierarchical case. The main drawback of MCMC is that when the posteriors’ structure is complex, with an increasing number of hierarchical levels and observations, the computation of the estimation process can become prohibitively expensive.

The earlier definition of the likelihood function and the prior distribution provides a general idea of how image processing can be realised under Bayesian modelling. In addition, it illustrates the potential ways of influencing posterior estimations, as prior distributions offer two options: an LMRF and a GMRF.

### 2.2 Sensitivity analysis for prior distribution

**Figure 1:**
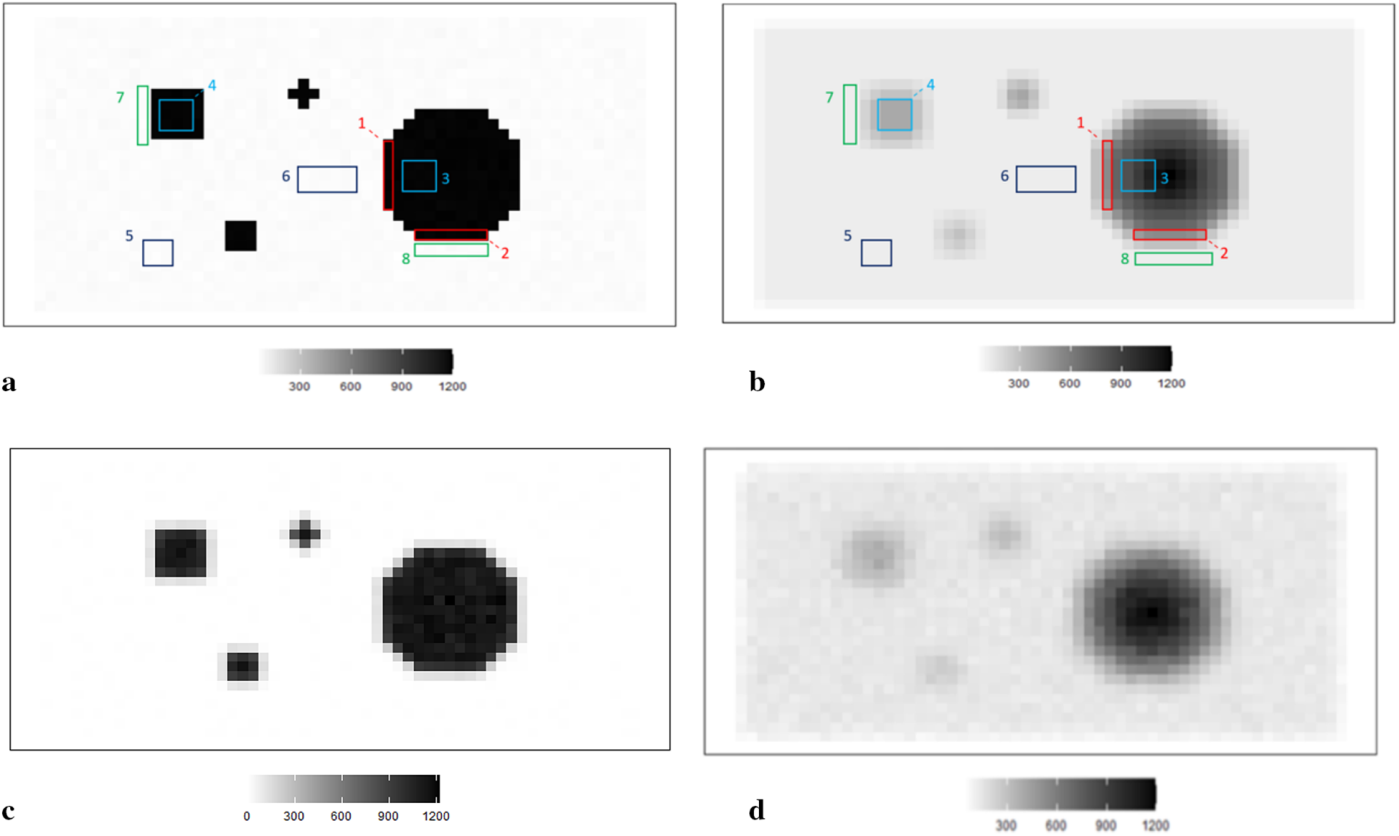
RoIs within different scanning experiences. **(a)** RoIs within the first simulation experience X, where there is a high contrast between the hot region and background. **(b)** RoIs within the second simulation experience $X_{1}$, where high-contrast edges are smoothing. **(c)** The observation of degraded image Y based on the simulation X. **(d)** The observation of degraded image $Y_{1}$ based on the simulation $X_{1}$.

In simulation applications, certain soft and high-contrast edges are intentionally designed for further estimation analysis, since the features of soft and high-contrast edges are essential for medical diagnosis in real-world clinical experience. In other words, detecting edges correctly can help identify tumors and other medical conditions. Hence, we employ two simulation datasets, as shown in Figure 1. For the first case, the average pixel value within the hot regions is around 1,099, while the average pixel value in the background is 0. For the second dataset, the pixels in the hot regions are 400, while the ones in the background are 75.

The simulated data is stored in a pixel matrix of size 29 × 58 and has been created as a potential truth for modelling outcomes assessment. One characterized by a high-contrast hot region, represented as **X**, and the other exhibiting smooth changes, denoted as **X**_1_. Observation **Y**, viewed as a degraded version of the actual image **X** with blur and noise, is the observation dataset comparable to the projection dataset in reality. As shown in Figure 1a, the actual image consists of four sharp regions with sharp boundaries. The largest is circular and located towards the right of the scan. The smallest is an irregular shape located at the bottom right of the circular. At the same time, the two other regions are both rectangular and located towards the left of the scanned image. Figure 1c depicts the observation image data with low contrast resolution; blur is evident around the edge of each region.

Supposing the objects have a soft edge instead of the hard one. In reality, a hard edge shown in Figure 1a would be challenging to detect. Instead, the edges are likely to be considerably more softer. Therefore, by applying a Gaussian kernel filter to the datasets presented in Figure 1b, we obtain a more smoothing set of data. If is the a Gaussian kernel, we say that. Similarly, the relationship between and is based on the Poisson likelihood function, as depicted in Figure 1d. The high blurring around the high-contrast edge between hot regions and the background makes it difficult to detect the original edge. The less helpful information could be used during the following reconstruction modelling.

#### 2.2.1 Regions of interest

Apart from the complete image as a globally estimated object, regions of interest (RoIs) within the image are distinguished by the corresponding location and the contrast in neighbouring values. ROIs in each simulation dataset are highlighted, as shown in Figures 1a,b accordingly.

The principle of identifying RoIs is based on the properties of pixel density. In our case, the smoothing area refers to small pixel variations, specifically those below 50. In high-contrast areas, the variation is much higher, for instance, above 500. Hence, the RoIs are labelled accordingly in Figure 1a. Furthermore, to investigate the estimation effects within the dataset with high smoothing levels, the same labels are applied in Figure 1b.

Regions 1 and 2 represent the high-contrast edges of the hot regions, where the density gradient is most pronounced. Examples of smoothed hot regions are denoted as Regions 3 and 4, indicating areas where the activity has evened out, whereas Regions 5 and 6 represent smoothing areas within the background, showing regions where the background density has been homogenised. Finally, instances of high-contrast edges in the background are highlighted in Regions 7 and 8, illustrating boundaries where there is a stark density difference in the cooler areas of the simulation.

#### 2.2.2 Homogeneous hyperprior parameter estimation

**Figure 2:**
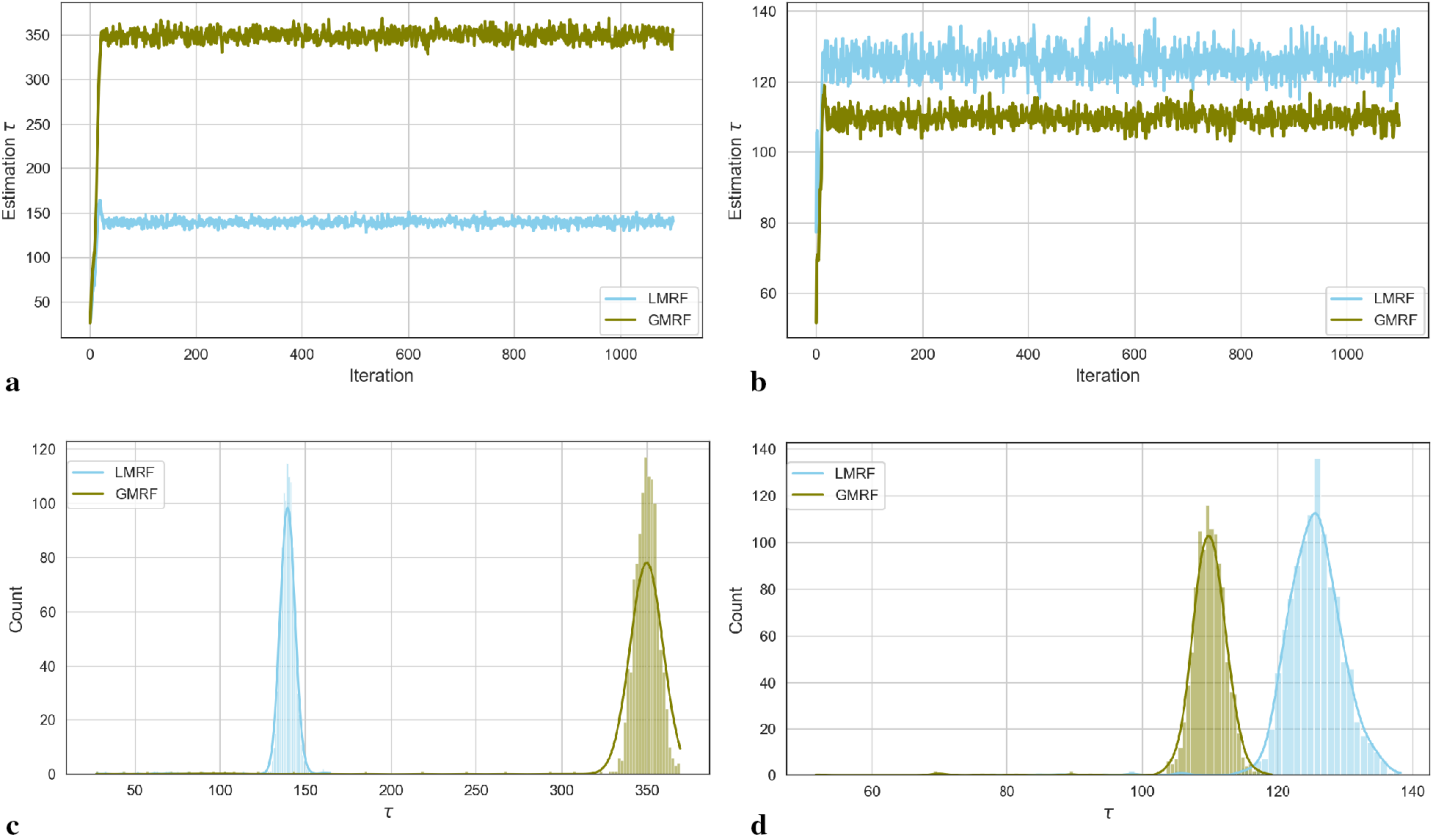
Estimation trace plots (top) and posterior distributions of hyperparameter τ for the first (left) and second (right) simulations under different priors. **(a)** Estimation trace plot of τ. **(b)** Estimation trace plot of τ. **(c)** Posterior distribution of τ. (**d**) Posterior distribution of τ.

For the two simulation examples, the homogeneous estimation for parameter τ after the introduction of hyperprior distribution π(τ)=1/τ is shown in the following convergent Monte Carlo chains in Figure 2. The trace plots demonstrate the convergence of the parameter estimation after a short burning period. As in, the first 100 samples are discarded as the chain reaches its stationary regime.

Based on the Goldilocks principle, one school believes that the acceptance rate should be in a range that is neither too high nor too low (20). Suppose the acceptance rate is high, indicating that the variance of the proposed value is small, almost every step can be accepted. In this case, obtaining a sample from every sample space using MCMC is computationally expensive as it requires a large number of iterations. However, if the acceptance rate is low, which results from using a large variance, almost every sample step will be rejected and the chain path will stick on a fixed figure. In addition, proposal jump sizes can be decreased as the low acceptance rate. Similarly, the size will increase as a high acceptance rate. The “0.234 rule” has been considered practically; proposing 0.234 is an asymptotically optimal acceptance rate (21). In other words, the sampling variance strongly depends on the comparison between 0.234 and the updated accept rate r in the current algorithm: for every 10 iterations, estimation $\tau^{\left(k+10\right)}=0.5\times \tau^{\left(k\right)}(1+r\times (1/0.234)))$. In our case, we continue to adopt this rule by scaling the proposal variance into the MCMC application to improve the efficiency of the algorithm.

In the case of the first simulation dataset, the estimated global hyperprior parameter τ is approximately 185 in the posterior distribution with a Laplace-type prior LMRF and a higher prior variance of τ=325 in the posterior distribution with a Gaussian-type prior GMRF, as illustrated in Figure 2a. For the second simulation application, the estimated value of τ in the LMRF is greater than the corresponding outcome in the GMRF, with values of approximately 110 and 130, respectively, as shown in Figure 2b. In addition, the posterior distributions of the hyperprior parameter in Figures 2c,d exhibit a symmetric Gaussian pattern, indicating the robustness of the estimations.

#### 2.2.3 Homogeneous prior parameter estimation

**Figure 3:**
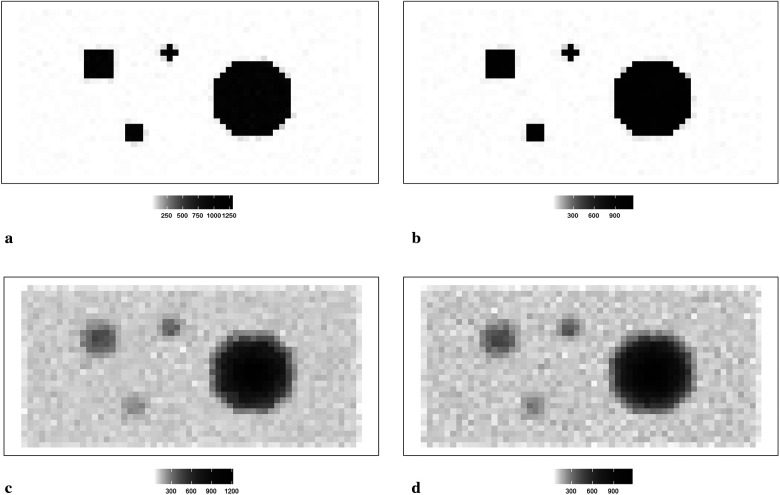
Image processing of two image simulation datasets under different prior distributions and optimum hypervariance τ. The two simulation datasets are denoted as I and II. Estimated images from a posterior distribution with Gaussian and Laplace random field priors denoted as GMRF and LMRF. **(a)** Estimated image from the model with GMRF I. **(b)** Estimated image from the model with LMRF I. **(c)** Estimated image from the model with GMRF II. **(d)** Estimated image from the model with LMRF II.

Here, we display the image estimate from the posterior estimation with an LMRF and a GMRF prior, accompanied by the globally optimum τ, as shown in Figure 3. There is more variation in the hot region from the left side compared with the right one in the first simulation application. For the second simulation dataset, the outcomes from both posterior distributions approach closely to the true value. The estimations indicate that both models can capture the underlying smoothing image patterns.

**Figure 4:**
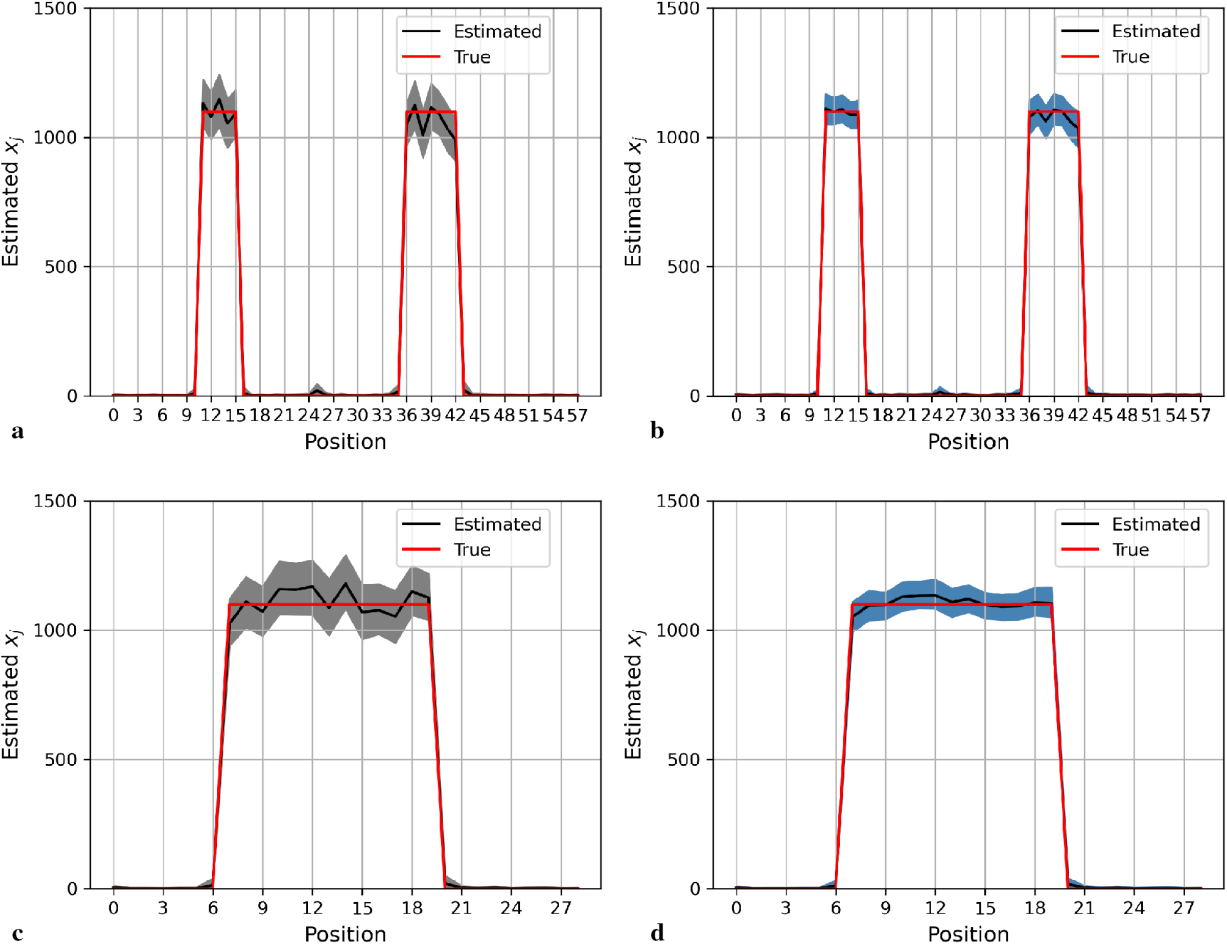
Posterior distributions of pixels under GMRF (left) and LMRF (right) prior distributions for the optimum homogeneous hyperprior variance parameter, employing the first simulation dataset. Here, the posterior estimations for the 20th row are shown at the top, whereas those for the 36th column are shown at the bottom. **(a)** Posterior estimation from the GMRF. **(b)** Posterior estimation from the LMRF. **(c)** Posterior estimation from the GMRF. **(d)** Posterior estimation from the LMRF.

Figure 4 shows the posterior estimation of pixels in the 20th row and the 36th column within the pixel matrix. The 20th row crosses two small hot regions and the 36th column crosses the largest circular hot region. Here, true pixel values are shown in red and pixel estimations from the different posterior distributions GMRF (left) and LMRF (right) are shown in grey and blue, respectively, accompanied by their associated confidence intervals. Both estimators from different priors approach the true pixel value. Although the confidence intervals for the two priors do cover the true values, the pixel posteriors for the LMRF over the hot regions are more uniform than those of the GMRF.

**Figure 5:**
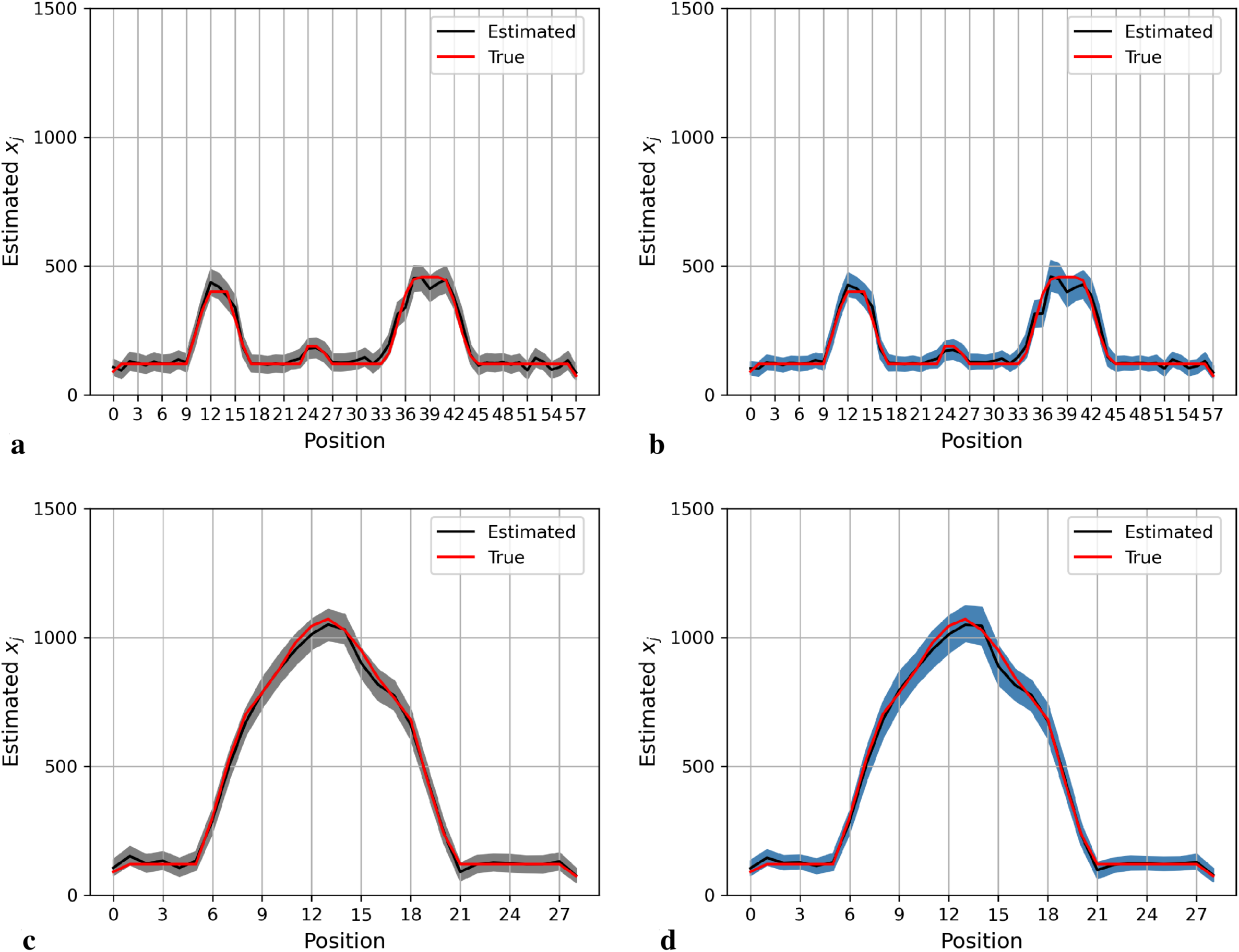
Posterior distributions of pixels under the GMRF (left) and LMRF (right) prior distributions for the optimum homogeneous hyperprior variance parameter, employing the second simulation dataset. Here, the posterior estimations for the 20th row are shown at the top and those for the 36th column are shown at the bottom. **(a)** Posterior estimation from the GMRF. **(b)** Posterior estimation from the LMRF. **(c)** Posterior estimation from the GMRF. **(d)** Posterior estimation from the LMRF.

Again, the posterior estimations of pixels within the 20th row and 36th column in the second simulation dataset are shown in Figure 5. The outcomes from both posterior distributions approach closely to the true value. The estimations indicate that both models can capture the underlying smoothing image patterns.

In general, the GMRF and LMRF posterior estimations are similar for the second simulation dataset, in terms of the pixel estimation $x_{j}$ and the homogeneous hyperprior parameter τ. It is opposed to the estimation conclusions from the first experience, in which the estimated homogeneous τ in the LMRF is smaller than those of the GMRF, and the variation within the estimation $x_{j}$ in the GMRF is higher than those of the LMRF.

#### 2.2.4 Estimation comparison within regions of interest

For the first estimation examples of the simulation dataset, the optimal τ obtained from the GMRF is larger than the one estimated from the LMRF, approximately 330 and 150, respectively. Similarly, the optimum τ from the GMRF for each RoI is also greater than the ones from the LMRF. Once τ exceeds the optimum value, the mean squared error (MSE) is experiencing a climb. It is noticeable that the MSE from the separate models overlap at the beginning and the end when τ is extremely small or large. In other words, improper τ may invalidate the effect of estimation from priors. When τ is large, say $O(10^{4})$, the LRMR and GRMR become non-informative priors, e.g., a uniform prior.

**Figure 6:**
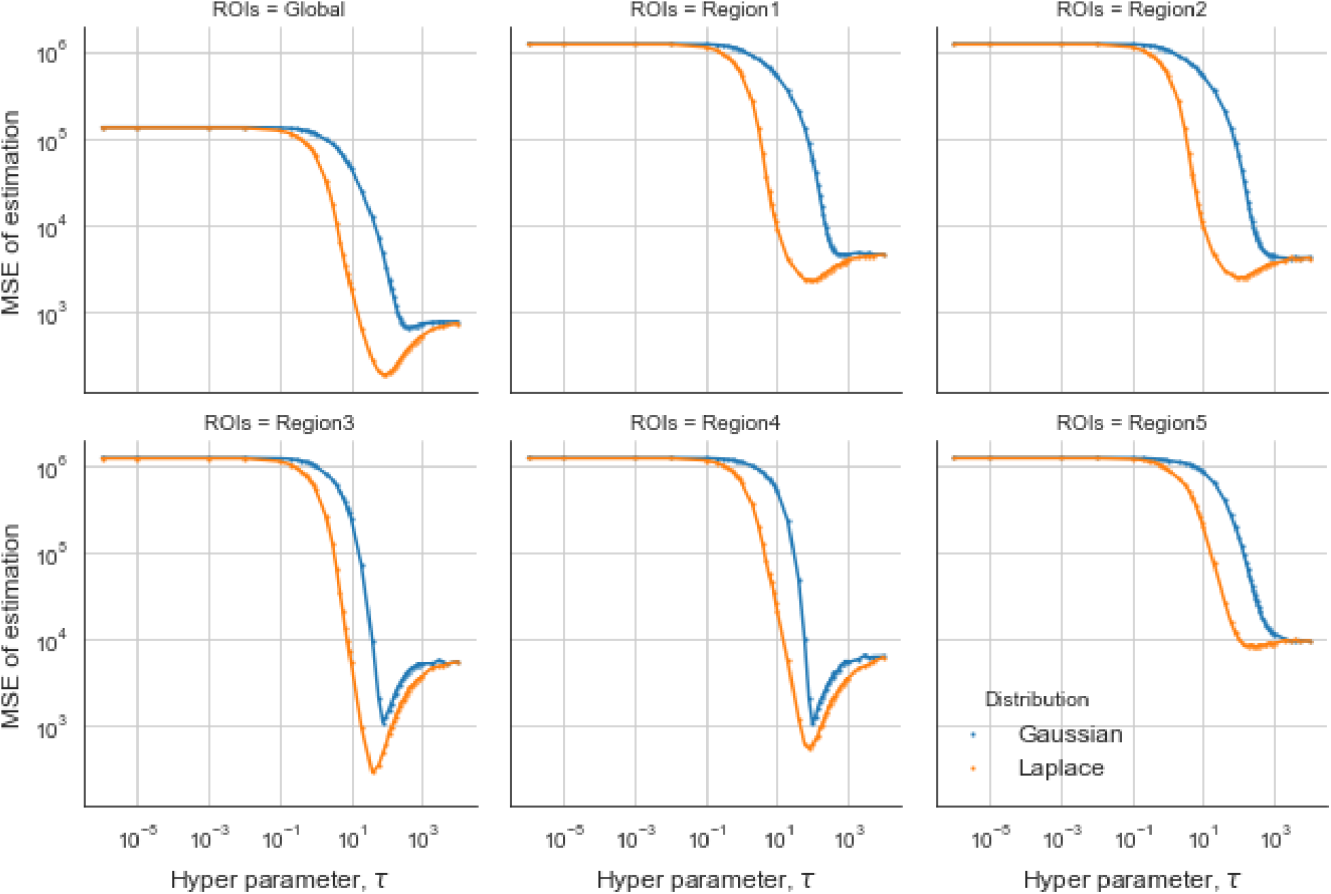
Estimation comparison between two models. Estimation comparison of different RoIs while varying hyperparameter τ. The blue lines indicate the MSE in the case of a Gaussian Markov random field prior, and the orange lines indicate the MSE in the case of a Laplace Markov random field prior.

**Figure 7:**
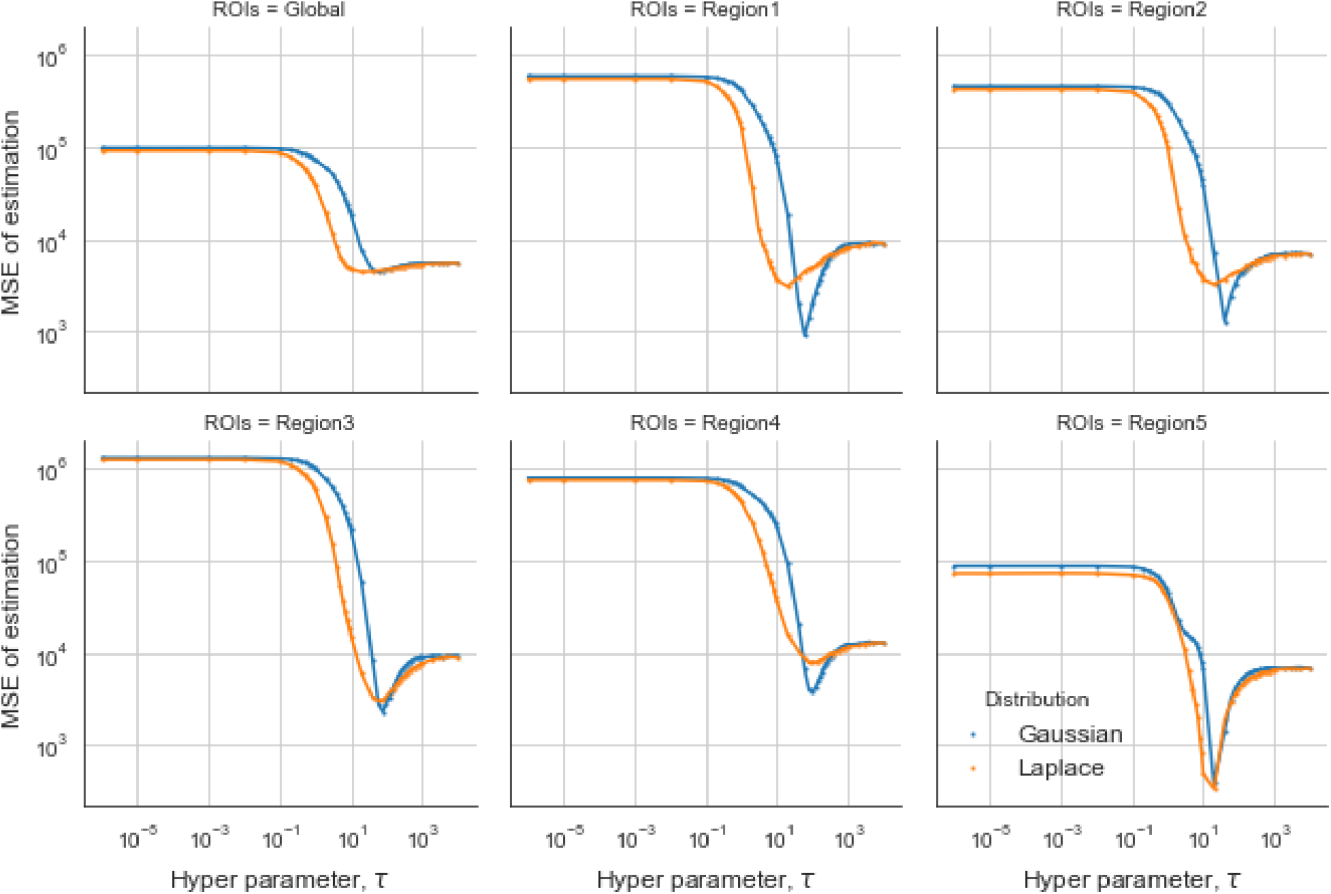
Estimation comparisons between two posterior distributions I. Estimation comparison of different RoIs while varying hyperparameter τ. The blue lines indicate the MSE in the case of a Gaussian Markov random field prior, and the orange lines indicate the MSE in the case of a Laplace Markov random field prior.

In the case of the LMRF, different optimum values of τ are obtained amongst the various RoIs when using the GMRF. Based on the MSE, the measurement compares the difference between the true value and the estimated value, providing the principle for our estimation comparisons. Figure 6 shows that the LMRF performs better both locally and globally compared with the GMRF. However, when referring to the second simulation dataset, the positions of the RoIs remain the same and the neighbouring pixels are smoother upon the application of a blur kernel. Figure 7, which compares the MSE of the two prior distributions while varying hyperparameter τ in different RoIs, shows that the LMRF prior performs better than that of the GMRF in RoIs 1 and 2. Although it shows that the global minimum MSE from the LMRF is slightly smaller than the one from the GMRF, the GMRF performs relatively better than the estimation from the LMRF in several RoIs, for instance, Regions 1 and 2. Based on the MSE performance, estimations from the GMRF are prior to the ones from the LMRF in terms of global estimation or the estimation within RoIs. Suppose there is another simulation dataset with a higher smoothing level than the first two, we can assume that GMRF can be an alternative solution for improving estimation accuracy when the neighbourhood is smooth.

## 3 Bayesian modelling with a mixture prior distribution

As pixels appear in different environments, for instance, smooth regions and high-contrast areas, prior distributions with different energy functions $\Phi_{j}(x)$ have accordingly different estimation effects. Therefore, we introduce a mixture prior distribution instead of a homogeneous prior into the application.

### 3.1 Mixture prior distribution

Assume the spatial information identifies a sharp boundary between hot regions and the background, represented by $\theta =\{\theta_{j},j=1,2,\ldots ,m\}$, which indicates a high-contrast edge between hot regions and the background. The binary element $\theta_{j}$ corresponds to $x_{j}$. In other words, if $\theta_{j}=0$, the pixel $x_{j}$ is more likely to be within a smooth environment (labelled by $\theta^{-}$). Otherwise, $x_{j}$ locates on high-contrast areas (labelled by θ) when $\theta_{j}=1$:

(9) $$\begin{array}{rl} \pi_{X|\theta ,\tau_{l},\tau_{g}}\left(x|\theta ,\tau_{l},\tau_{g}\right)= & \frac{1}{Z}\prod_{\;j=1}^{m}(\frac{\theta_{\;j}}{2\tau_{l}}\exp\left(-\frac{\sum_{t\in \partial \left(\;j\right)}|x_{j}-x_{t}|}{\tau_{l}}\right)\\ & +\frac{\left(1-\theta_{j}\right)}{\sqrt{2\pi }\tau_{g}}\exp\left(-\frac{\sum_{t\in \partial \left(\;j\right)}{\left(x_{j}-x_{t}\right)}^{2}}{2\tau_{g}^{2}}\right)), \end{array}$$

where $X=\{x_{j},\;j=1,2,\ldots ,m\}$, $\theta =\{\theta_{j},\;j=1,2,\ldots ,m\}$, and $\theta_{j}$ is a binary variable that has two values; either $\theta_{\;j}=1$ or $\theta_{\;j}=0$. The hyperprior variances in the LMRF and GMRF priors are denoted as $\tau_{l}$ and $\tau_{g}$, respectively.

### 3.2 Assignment of hyperprior parameters

In medical imaging, the recording signal contrast between the tissues varies by scanning time, the radioactive tracer, the type and amount of tissues, and the post-processing technique. It is difficult to estimate the pixel differences in different scenarios. Hence, the assignment for hyperparameters $\tau_{l}$ and $\tau_{g}$ within the Bayesian model is required to be capable of capturing variation within pixels and realise the robust improvement in estimation accuracy. In addition, the mixture of prior distributions within the Bayesian model should be distinguished. Otherwise, the modelling cannot classify the different terms of pixels into two basic scenarios (small- and high-value variation). It is known that the Bayesian model with the GMRF prior performs better regarding smooth areas. For defining distributions for the smoothing area, we can assign small expected variance ${\hat{\tau }}_{g}=10$ and the other prior distribution with expected ${\hat{\tau }}_{l}=100$ in the Laplace random field prior. Therefore, we introduce Gaussian hyperprior distributions for $\tau_{l}$ and $\tau_{g}$ with their mean parameters equal to 100 and 10, respectively, and the standard variance equal to 1:

(10) $$\pi \left(\tau_{l}\right)=\frac{1}{\sqrt{2\pi }}\exp\left(-|\tau_{l}-100{|}^{2}\right);\;\pi \left(\tau_{g}\right)=\frac{1}{\sqrt{2\pi }}\exp\left(-|\tau_{g}-10{|}^{2}\right).$$

The primary hypothesis we hold is that the external spatial information θ is not available, which requires the introduction of another probability ρ to decide the most likely hyperprior distributions for each pixel. Therefore, the spatial location $\theta_{j}$ for each pixel would have two results—either the pixel is within the high-contrast area with a probability ρ or within the smoothing area with a probability 1−ρ. Finally, the spatial factor $\theta =\{\theta_{\;j},\;j=1,2,3,\ldots ,m\}$ is the collection for the whole event. It is a conditional Bernoulli distribution based on the probability $\pi_{\theta |\rho }(\theta |\rho )$:

(11) $$\pi_{\theta |\rho }\left(\theta |\rho \right)\propto \prod_{\;j=1}^{m}\rho^{\theta_{\;j}}(1-\rho {)}^{1-\theta_{\;j}},\;\rho >0.$$

### 3.3 Hyperprior distribution

For the hyperprior distribution of the probability parameter ρ, inside the hyperprior distribution π(θ∣ρ), the beta distribution with parameters α and β is considered. As the beta distribution is a conjugate prior distribution, and the value range of the variable ρ is between 0 and 1, it is suitable for representing probabilities. The expression for the beta hyperprior distribution is as follows:

(12) $$\rho ∼\;\text{beta}\left(\alpha ,\beta \right);\;\pi_{\rho |\alpha ,\beta }\left(\rho |\alpha ,\beta \right)\propto \frac{\rho^{\alpha -1}{\left(1-\rho \right)}^{\beta -1}}{B\left(\alpha ,\beta \right)};\;\alpha >0,\beta >0,$$

where ρ∈[0,1], β, and α are positive parameters within the beta distribution $\pi_{\rho |\alpha ,\beta }(\rho |\alpha ,\beta )$.

When the shape parameter α and rate parameter β in the beta distribution both equal 0.5, there are two peaks in the density function located at the boundaries of the parameter space; in other words, the probabilities when ρ=0 and ρ=1 are higher than other values of ρ. This U-shaped distribution can help classify a pixel into two different prior distributions: the prior distribution with an absolute energy function (LMRF) and the prior distribution with a squared energy function (GMRF).

The posterior distribution is obtained after multiplying all the defined terms:

(13) $$\begin{array}{rl} \pi_{X,\tau_{l},\tau_{\;j}|\theta }\left(x,\tau_{l},\tau_{g}|\theta \right) & =\pi_{X|\theta ,\tau_{l},\tau_{\;j}}\left(x|\theta ,\tau_{l},\tau_{g}\right)\pi \left(\tau_{l}\right)\pi \left(\tau_{g}\right)\\ & \;=\prod_{\;j=1}^{m}(\frac{\theta_{\;j}}{2\tau_{l}}\exp\left(-\frac{\sum_{t\in \partial j}|x_{j}-x_{t}|}{\tau_{l}}\right)\frac{1}{\sqrt{2\pi }}\exp\left(-|\tau_{l}-100{|}^{2}\right)\\ & \;\;+\frac{\left(1-\theta_{j}\right)}{\sqrt{2\pi }\tau_{g}}\exp\left(-\frac{\sum_{t\in \partial j}{\left(x_{j}-x_{t}\right)}^{2}}{2{\tau_{g}}^{2}}\right)\frac{1}{\sqrt{2\pi }}\exp\left(-|\tau_{g}-10{|}^{2}\right)). \end{array}$$

**Figure 8:**
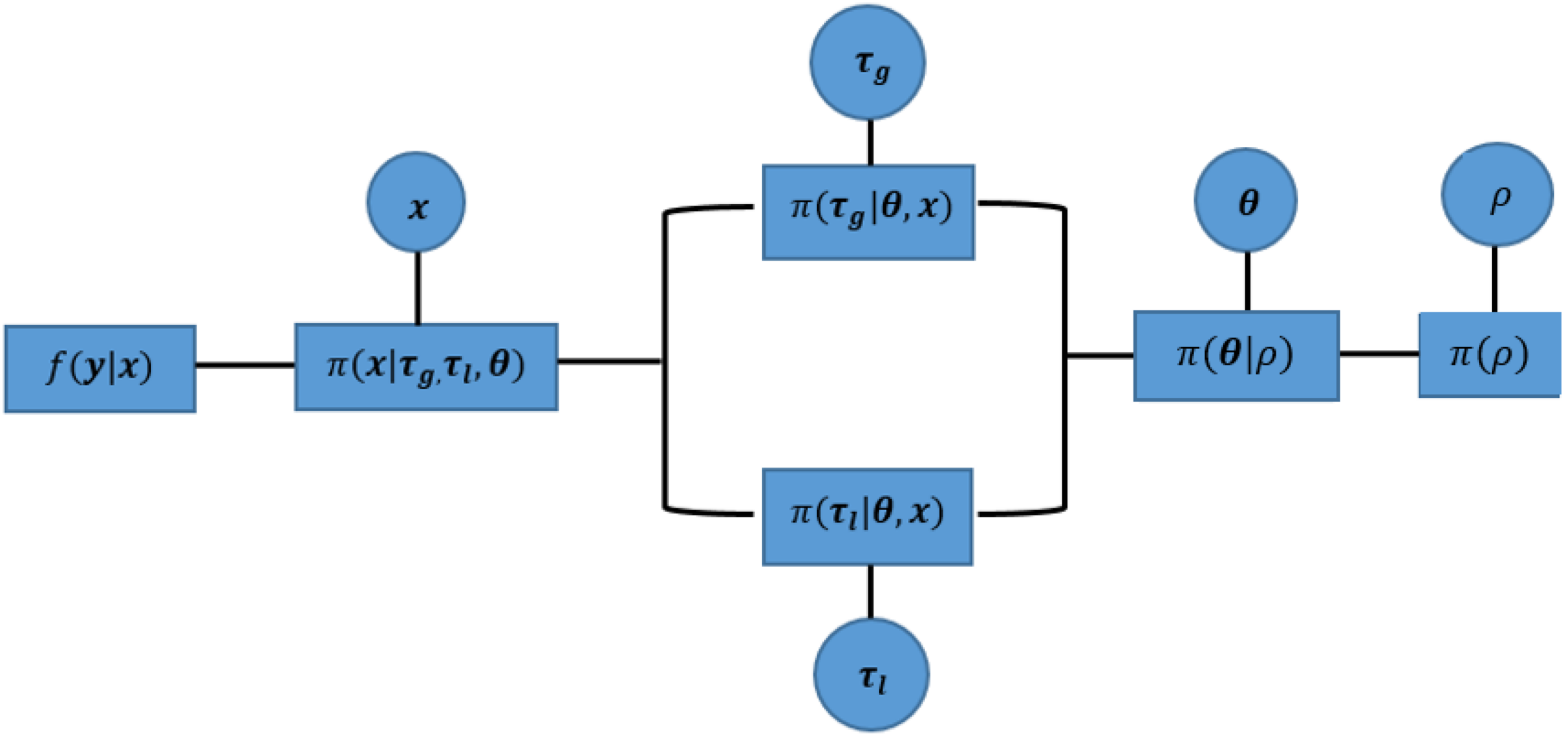
Parallel implementation of a sequential Markov chain in Monte Carlo simulations. The components within this hierarchical Bayesian modelling from left to right are the likelihood function between observation Y and the true unknown pixels X, mixture prior distribution for X, hyperprior distribution of variances $\tau_{g}$ and $\tau_{l}$, hyperprior distribution of spatial factor θ, and the hyperprior distribution for probability ρ. Circles represent the unknown parameters for further estimation, and squares indicate the correspondingly defined distribution. In addition, the corresponding estimation process of MCMC can be found in Table 1.

Within a hierarchical Bayesian model, the estimation of unknown parameters follows a sequential order from the bottom level of prior parameters to the highest level of hyper parameters. This sequential process still applies to our MCMC approach, apart from the parallel estimation for hyperparameters $\tau_{l}$ and $\tau_{g}$. Since θ, as a prior selection factor, allocates estimates into two hyperprior distributions, the corresponding hypervariance parameters $\tau_{l}$ and $\tau_{g}$ are conditional independent. Both parameters can be estimated simultaneously before the estimation process moves to the next stage, as seen in Figure 8.

**Table 1:** MCMC for modelling with locally adaptive hyperprior parameter ρ.

**Algorithm** MCMC for modelling with locally adaptive hyper prior distribution
For iteration k
**Input**: A list of initial values $\left\{X_{1}=x_{1}^{\left(0\right)},\;X_{2}=x_{2}^{\left(0\right)},\;\ldots ,\;X_{m}=x_{m}^{\left(0\right)}\right\}$;
$\left\{\theta_{1}=\theta_{1}^{\left(0\right)},\;\theta_{2}=\theta_{2}^{\left(0\right)},\;\ldots ,\;\theta_{m}=\theta_{m}^{\left(0\right)}\right\}$;
An initial positive constant $\rho^{0}$.
**For** j={1,2,…,m}
1. Propose a new value $x_{\;j}^{\left(k\right)}∼N(x_{\;j}^{\left(k-1\right)},{\left(\sigma^{\left(k\right)}\right)}^{2})$; **if and only if $x_{\;j}^{k}\geq 0$**
2. Generate μ∼unif(0,1)
3. Accept $x_{j}^{k}$ with probability
$\alpha =\text{min}(1,\frac{\;f_{X|Y,\theta ,\tau_{g},\tau_{l}}(x_{1}^{\left(k-1\right)},x_{2}^{\left(k-1\right)},\ldots ,x_{\;j}^{\left(k\right)},\ldots ,x_{m}^{\left(k-1\right)}|y,\theta ,\rho^{\left(k-1\right)})}{f_{X|Y,\theta ,\tau_{g},\tau_{l}}(x_{1}^{\left(k-1\right)},x_{2}^{\left(k-1\right)},\ldots ,x_{\;j}^{\left(k-1\right)},\ldots ,x_{m}^{\left(k-1\right)}|y,\theta ,\rho^{\left(k-1\right)})})$
4. Compare the μ with the calculated α,
5. **if**: μ≤α **then**
6. Accept the proposal value $x_{j}=x_{\;j}^{\left(k\right)}$
7. **else** $x_{j}=x_{\;j}^{\left(k-1\right)}$
**end** updating x
Updating bivariate θ follows similar steps between 1 to 7 but with a different hyperprior distribution.
8. Propose a new candidate value $\rho^{k}∼N(\rho^{k-1},(\sigma_{\rho }^{\left(k\right)}{)}^{2})$; **if and only if $\rho^{k}\geq 0$**
9. Generate $\mu_{\rho }∼\text{unif}(0,1)$.
10. Accept $\rho^{\left(k\right)}$ with probability:
$\alpha_{p}\rho =\text{min}(1,\frac{\;f_{\rho |X,\theta ,\tau_{l},\tau_{g}}(\rho^{\left(k\right)}|x^{k},\theta^{k})}{f_{\rho |X,\theta ,\tau_{l},\tau_{g}}(\rho^{\left(k-1\right)}|x^{k},\theta^{k})}),$
**if**: $\mu_{\rho }\leq \alpha_{\rho }$ **then**
11. Accept the proposal value $\rho =\rho^{\left(k\right)}$,
**else** $\rho =\rho^{\left(k-1\right)}$
12. **end if**
**end** updating ρ
Repeat the above steps until receiving a sufficiently large sampling size.

## 4 Posterior estimation from the Bayesian model

The spatial information describes the variation within the surroundings of a pixel. If spatial information is available prior to image processing, it can help distinguish the sub-regions based on particular features, such as high contrast and blurred. In particular, these features can help determine which modelling process should be used. The posterior estimation for classification label θ and unknown image X can be estimated by locally adaptive Bayesian modelling with a conjugate beta prior distribution. The shape α and rate β parameters in the beta prior distribution are both equal to 0.5, and the Bayesian model with the single prior distribution with an absolute energy function (LMRF) is regarded as the corresponding comparison.

### 4.1 Posterior estimation comparison

**Figure 9:**
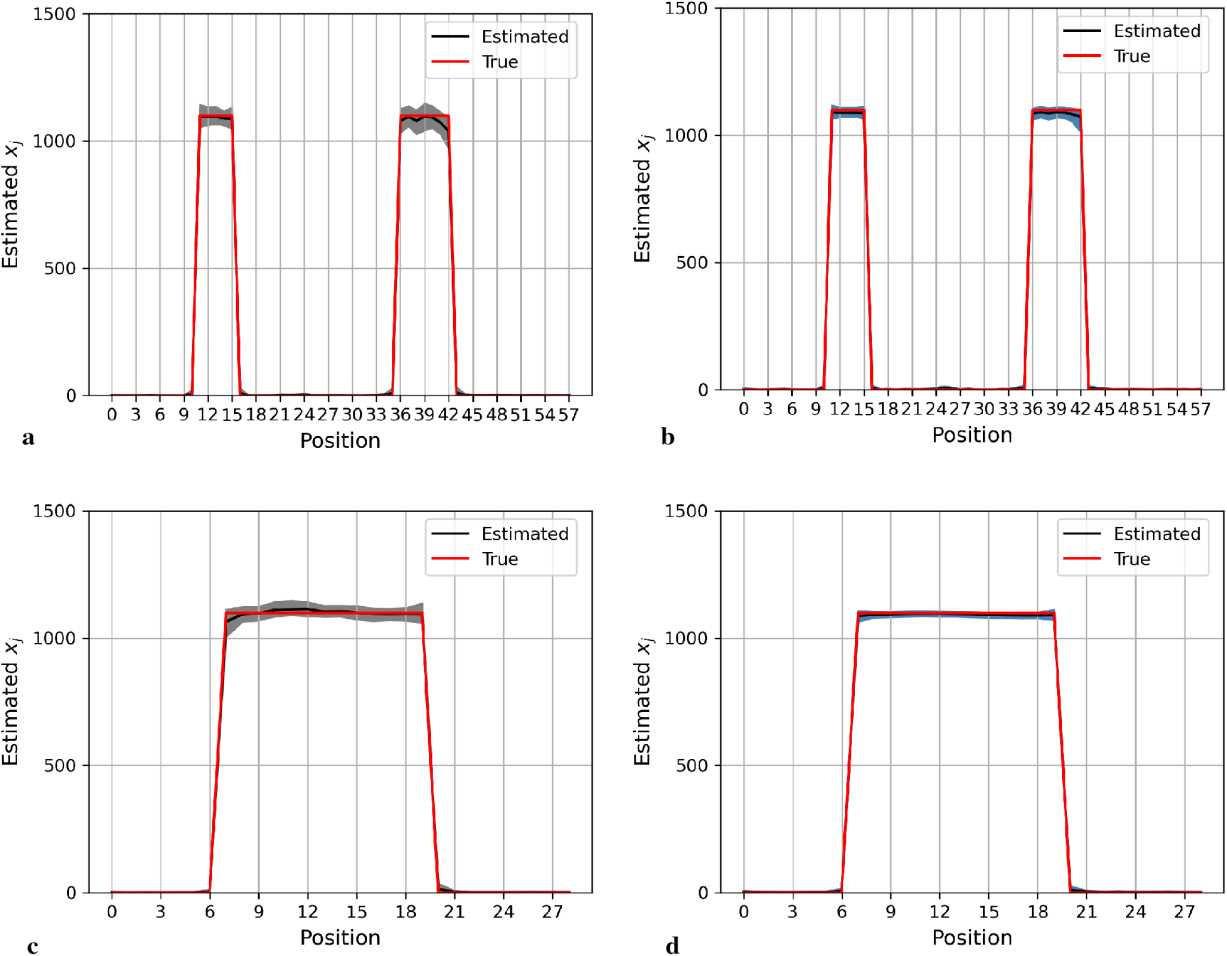
Posterior estimations of pixels with mixture prior distribution in the first simulation. Here, the posterior estimations for the 20th row are shown at the top, and those for the 36th column are shown on the bottom. **(a)** and **(c)** show the pixel estimations from a posterior distribution with a homogeneous hyperprior variance using an LMRF. **(b)** and **(d)** show the pixel estimations from a posterior distribution with a locally adaptive mixture prior distribution. **(a)** Homogeneous prior distribution. **(b)** Mixture prior distribution. **(c)** Homogeneous prior distribution. **(d)** Mixture prior distribution.

**Figure 10:**
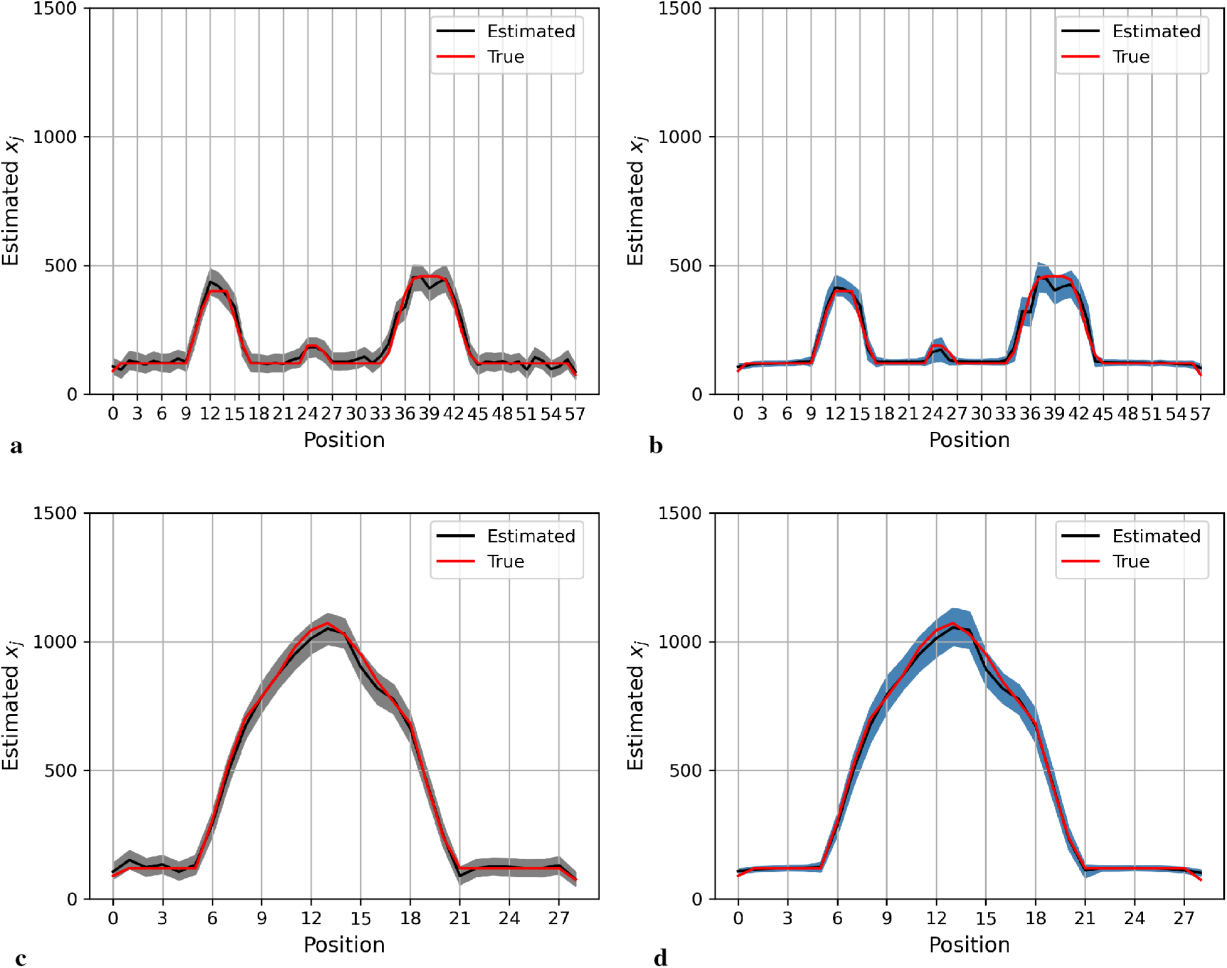
Posterior estimations of pixels with mixture prior distribution in the second simulation. Here, the posterior estimations for the 20th row are shown at the top, and those for the 36th column are shown on the bottom. **(a)** and **(c)** show the pixel estimations from a posterior distribution with a homogeneous hyperprior variance using an LMRF. **(b)** and **(d)** show the pixel estimations from a posterior distribution with a locally adaptive mixture prior distribution. **(a)** Homogeneous prior distribution. **(b)** Mixture prior distribution. **(c)** Homogeneous prior distribution. **(d)** Mixture prior distribution.

Here, we present the posterior estimation derived from a posterior distribution that combines multiple prior distributions. In addition, we provide posterior estimation results using a posterior distribution that incorporates the single prior distribution with the LMRF for comparison. The optimal hyperprior variance with the LMRF is obtained in Section 2.2.2. As seen in Figures 9 and 10, the estimate from the posterior distribution with mixture prior distribution (right) has less variation than the estimate with the locally adaptive hyperprior parameter (left). Furthermore, the MSE is reduced after employing the posterior distribution with the mixture prior distribution.

In the first simulation application, when estimating pixels from the posterior distribution with a mixture prior, the credible intervals for posterior estimates within the hot regions are more stable than those derived from the posterior distribution with a homogeneous prior distribution, as seen in Figure 9. For the second simulation application, as seen in Figure 10, the estimation performance from both posterior distributions is quite similar. Both posterior distributions can realise image deblurring and denoising.

### 4.2 Real application in small animal imaging

**Figure 11:**
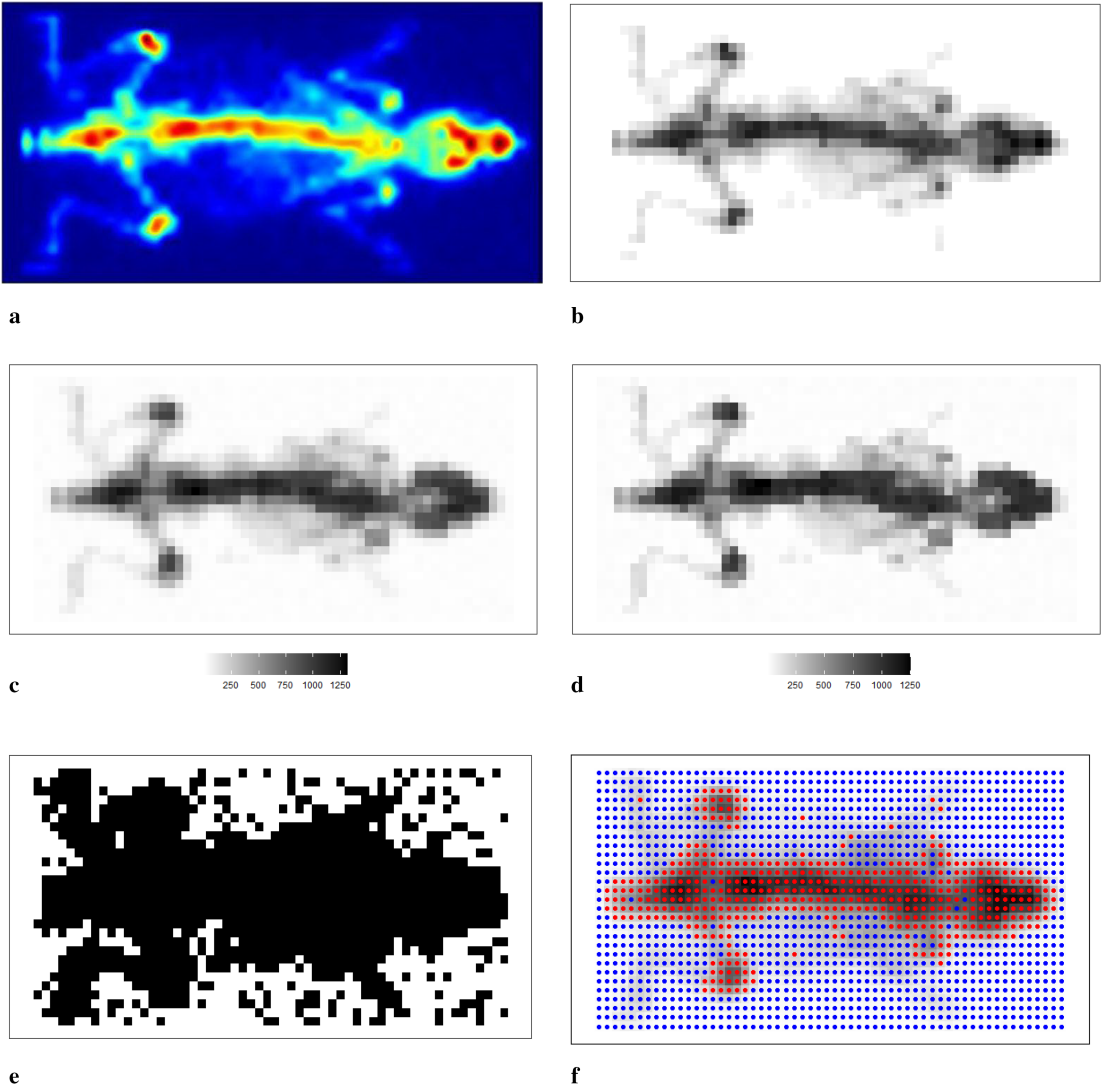
Scan of a mouse using $\gamma {\text{-eye}}^{\text{TM}}$. **(a**–**c)** The real scan of the mouse, a correspondingly simulated dataset and degraded observation dataset, respectively; **(d)** represents the posterior estimations from Bayesian modelling; **(e**,**f)** the estimated bivariate spatial factors and classification outcomes by using *k*-means. **(a)** Mouse scan using $\gamma {\text{-eye}}^{\text{TM}}$. **(b)** Simulated image derived from the true scan. **(c)** Degraded observation image. **(d)** Estimated image from the application. **(e)** Estimated classification. **(f)** Classification from *k*-means.

We now apply the Bayesian model with mixture prior distributions to medical images obtained from mouse scans using $\gamma {\text{-eye}}^{\text{TM}}$ to confirm the conclusions obtained from the previous sections. Figure 11a shows the image of a mouse (22) injected with Tc99m labelled radiotracer acquired with $\gamma {\text{-eye}}^{\text{TM}}$, and Figure 11b presents a correspondingly designed dataset for assessment of the estimation procedure. Bayesian modelling with a mixture prior distribution estimates the true image based on the degraded observation image with additional noise and blurring, as depicted in Figure 11c. The posterior estimate of the underlying radiotracer activity in Figure 11d demonstrates a significant improvement in image quality.

In Bayesian modelling, the binary hyperprior parameter of the spatial factor θ determines the prior distribution of a pixel based on its corresponding neighbourhoods. Pixels $x_{j}$ are classified within the hot regions when $\theta_{j}$ equals 1. However, when $\theta_{j}$ equals 0, there is a high probability that the pixel is within the smoothing region, especially the background. We present the image pattern of spatial factor θ in Figure 11e. From the image, it is evident that the spatial factor can effectively classify pixels within different environments, such as hot regions and the background. In addition, the edge between the hot regions and the background is easily detected. Here, the classification outcome from *k*-means^1^ is presented as a comparison with the classification from the hyperprior parameter θ, as seen in Figure 11f. The classification method of *k*-means successfully identifies the two clusters of pixels: one containing the high pixel value and the other containing the low pixel value. However, this method fails to classify the pixels that have a relatively small value but are within the hot regions. The edge detection between hot regions and the background is not as accurate when compared with the classification results obtained from Bayesian modelling.

## 5 Conclusion

The Bayesian approach shows the advantage of estimating unknown parameters without the need for a big data environment. In addition, Bayesian estimation can provide high-quality medical images by deblurring and denoising. For sensitivity analysis of Bayesian prior distributions, the estimation object includes not only the completed image as a single observation but also several pixel segments based on different pixel neighbourhoods, such as high-contrast edges and smoothing areas. The latter refers to local sensitivity analysis, revealing that locally adaptive selection for prior distributions with dissimilar properties can be one of the solutions for improving estimation accuracy.

In our application, the mixture prior distribution comprises two MRF priors with distinct energy functions: $\Phi_{j}(x)=\sum_{t\in \partial (j)}|x_{j}-x_{t}|$ and $\Phi_{j}(x)=\sum_{t\in \partial (j)}(x_{j}-x_{t}{)}^{2}$. The spatial factor, denoted as $\theta =\{\theta_{j},j=1,2,\ldots ,m\}$, is embedded within the mixture prior distribution as a bivariate label, determining the pixels’ prior distribution. Furthermore, the spatial factor as a hyperprior parameter not only contributed to the prior distribution of pixels but also provided the classification information. In other words, our spatial factor classified the pixel environments into two clusters, identifying one as smooth areas and the other as high-contrast areas. This clustering function is analogous to classification methods in machine learning, predicting the probability of the occurrence of a binary outcome.

Although the initial application dataset presents a two-dimensional image, when transforming the dataset from projection images to tomography images, each pixel in the two-dimensional space corresponds to a voxel in three-dimensional space. This transformation allows us to introduce two additional neighbours for each pixel, based on the first-order system. Furthermore, considering time as a variable in the model enables its application to high-dimensional datasets.

## Data Availability

The raw data supporting the conclusions of this article will be made available by the authors, without undue reservation.

## Footnotes

^1^The corresponding algorithm for *k*-means can be found in the Appendix.

## Appendix

### List of Notation

$X=\{X_{1},X_{2},\ldots ,X_{m}\}$: the observed object consisting of unknown elements that cannot be obtained directly.

$P=\{P_{1},P_{2},\ldots ,P_{n}\}$: the projection dataset that contains information detected about X by different imaging techniques.

$Y=\{Y_{1},Y_{2},\ldots ,Y_{n}\}$: the simulated projection dataset based on some simulated truth after adding artificial noise.

$\tau =\{\tau_{1},\tau_{2},\ldots ,\tau_{m}\}$: the set that contains the local variation parameters in a hyperprior distribution.

$\tau ,\tau_{l},\tau_{g}$: the global hyperprior variance according to different scenarios.

$\tau^{k}$: the kth estimation of τ in MCMC.

$\rho =\{\rho_{1},\rho_{2},\ldots ,\rho_{m}\}$: the set of hyperprobabilities in a hyperprior Bernoulli distribution.

$\theta =\{\theta_{j},j=1,2,\ldots ,m\}$: the set of locally spatial factors in a mixture prior distribution.

$z=\left(z_{j},j=1,2,\ldots ,m\right)$: the external dataset containing the spatial information.

$\Phi_{j}\left(x\right)=\sum_{t\in \partial \left(\;j\right)}w_{\;jt}\phi \left(x_{j}-x_{t}\right)$: the local energy function between targeted $x_{j}$ and its four closest neighbours $x_{t,t\in \partial \left(\;j\right)}$.

$\phi \left(x_{j}-x_{t}\right)$: the potential function of targeted pixel $x_{j}$.

$w_{\;jt}$: the weight for each paired comparison of pixels.

π(γ|ξ,θ): a conjugate hyperprior distribution of γ-Gamma distribution with shape parameter ξ and scale parameter θ.

$A=\left\{\begin{array}{ccc} a_{11} & \ldots & a_{1m}\\ \vdots & \ddots & \vdots \\ a_{n1} & \ldots & a_{nm} \end{array}\right\}$: the transformation matrix that comprises probability $a_{ij}$ of information $X_{j}$ corresponding to the observation dataset.

### *k*-means application

Assigning a critical value subjectively poses challenges. Therefore, an alternative solution involves estimating external information through supervised classification methods, such as *k*-means clustering. In this approach, a measurement matrix is designed to include pixel indices, individually estimated values, and the four differences with neighbours in the first system (up, down, left, and right directions). Subsequently, *k*-means clustering is applied to classify pixels based on these indices. The structure of the index table is as follows:

The estimation value for pixel is denoted as $x_{\;j}\;:\;j=\{1,2,3,\ldots ,m\}$ and ${\hat{x}}_{t\in \partial (j)}=\{{\hat{x}}_{t1},{\hat{x}}_{t2},{\hat{x}}_{t3},{\hat{x}}_{t4}\}$ is the collection of four-direction neighbours. Each pair of differences can also be calculated as the estimation difference. Assuming pixels in hot regions and the background are treated as two clusters, the intersections between the two clusters are considered high-contrast grids.

### Measurement of estimation

Table A2 shows the corresponding estimation measurements of six selected pixels in the 20th row in the first simulation application. Overall, the Bayesian model with mixture prior distribution introduces estimation flexibility to realise a more accurate outcome in each application.

**Table A1:** Measurement indices for clustering.

Pixel	Estimation	Up	Down	Left	Right
$x_{j}$	${\hat{x}}_{j}$	$\left|{\hat{x}}_{j}-{\hat{x}}_{t1}\right|$	$\left|{\hat{x}}_{j}-{\hat{x}}_{t2}\right|$	$\left|{\hat{x}}_{j}-{\hat{x}}_{t3}\right|$	$\left|{\hat{x}}_{j}-{\hat{x}}_{t4}\right|$

**Table A2:** The list includes estimation measurements.

Position	lci.M	uci.M	mean.M	lci.H	uci.H	mean.H
37	1.06e+03	1,105.94	1,084.85	1.03e+03	1,125.54	1,078.53
38	1.07e+03	1,111.14	1,089.67	1.06e+03	1,136.83	1,094.03
39	1.06e+03	1,106.67	1,085.18	1.02e+03	1,119.64	1,078.34
40	1.07e+03	1,109.43	1,089.67	1.04e+03	1,146.91	1,095.90
41	1.07e+03	1,108.75	1,088.11	1.05e+03	1,136.12	1,090.97
42	1.05e+03	1,104.03	1,080.87	1.03e+03	1,111.63	1,071.76

“H” indicates the posterior estimation from Bayesian modelling with the global LMRF, and “M” indicates the posterior estimation from Bayesian modelling with a locally adaptive mixture prior distribution. The outcomes are stored to two decimal places. “lci” and “uci” represent the lower credible interval and upper credible interval, which indicate the range within which a parameter lies with 95% probability.

## References

[1] Kasban H El-Bendary M Salama D. A comparative study of medical imaging techniques. Int J Inf Sci Intell Syst. (2015) 4:37–58.

[2] Kastis GA Furenlid LR Wilson DW Peterson TE Barber HB Barrett HH. Compact CT/SPECT small-animal imaging system. IEEE Trans Nucl Sci. (2004) 51:63–7. 10.1109/TNS.2004.823337 26538684 PMC4629807

[3] Kastis GA Wu MC Balzer SJ Wilson DW Furenlid LR Stevenson G, et al. Tomographic small-animal imaging using a high-resolution semiconductor camera. IEEE Trans Nucl Sci. (2002) 49:172–5. 10.1109/TNS.2002.998747 PMC4643294 26568676

[4] Tsoumpas C Visvikis D Loudos G. Innovations in small-animal PET/MR imaging instrumentation. PET Clin. (2016) 11:105–18. 10.1016/j.cpet.2015.10.005 26952725

[5] Katartzis A Petrou M. Current trends in super-resolution image reconstruction. Image Fus Algorithms Appl. (2008) 1:1–26. 10.1016/B978-0-12-372529-5.00007-X

[6] Winkler G, Image Analysis, Random Fields and Markov Chain Monte Carlo Methods. Berlin: Springer (2003).

[7] Karaoglanis K Polycarpou I Efthimiou N Tsoumpas C. Appropriately regularized OSEM can improve the reconstructed PET images of data with low count statistics. Hell J Nucl Med. (2015) 18(2):140–5. 10.1967/s002449910209 26187214

[8] Maestrini L Aykroyd RG Wand MP. A variational inference framework for inverse problems. *arXiv:2103.05909* (2021). Available online at: https://arxiv.org/abs/2103.05909.

[9] Kukačka J Metz S Dehner C Muckenhuber A Paul-Yuan K Karlas A, et al. Image processing improvements afford second-generation handheld optoacoustic imaging of breast cancer patients. Photoacoustics. (2022) 26:100343. 10.1016/j.pacs.2022.100343 35308306 PMC8931444

[10] Varrone A Sjöholm N Eriksson L Gulyás B Halldin C Farde L. Advancement in PET quantification using 3D-OP-OSEM point spread function reconstruction with the HRRT. Eur J Nucl Med Mol Imaging. (2009) 36:1639–50. 10.1007/s00259-009-1156-3 19437012

[11] Voskoboinikov YE. A combined nonlinear contrast image reconstruction algorithm under inexact point-spread function. Optoelectron Instrum Data Process. (2007) 43:489–99. 10.3103/S8756699007060015

[12] Deidda D Karakatsanis NA Robson PM Efthimiou N Fayad ZA Aykroyd RG, et al. Effect of PET-MR inconsistency in the kernel image reconstruction method. IEEE Trans Radiat Plasma Med Sci. (2018) 3:400–9. 10.1109/TRPMS.2018.2884176 33134651 PMC7596768

[13] Marsi S Bhattacharya J Molina R Ramponi G. A non-linear convolution network for image processing. Electronics. (2021) 10:201. 10.3390/electronics10020201

[14] Matej S Fessler JA Kazantsev IG. Iterative tomographic image reconstruction using Fourier-based forward and back-projectors. IEEE Trans Med Imaging. (2004) 23:401–12. 10.1109/TMI.2004.824233 15084066

[15] Winkler G, Image Analysis, Random Fields and Markov Chain Monte Carlo Methods: A Mathematical Introduction. Vol. 27. Berlin: Springer Science & Business Media (2012).

[16] Al-Gezeri SM Aykroyd RG. Spatially adaptive Bayesian image reconstruction through locally-modulated Markov random field models. Braz J Probab Stat. (2019) 33:498–519. 10.1214/18-BJPS399

[17] Geman S Geman D. Stochastic relaxation, Gibbs distributions, and the Bayesian restoration of images. IEEE Trans Pattern Anal Mach Intell. (1984) PAMI-6(6):721–41. 10.1109/TPAMI.1984.4767596 22499653

[18] Nikou C. MAP tomographic reconstruction with a spatially adaptive hierarchical image model. In *2017 25th European Signal Processing Conference (EUSIPCO)*. IEEE (2017). p. 1549–53. 10.23919/EUSIPCO.2017.8081469

[19] DeGroot D Lindstrom G, Logic Programming: Functions, Relations, and Equations. Prentice-Hall, Inc. (1986).

[20] Somero GN. The goldilocks principle: a unifying perspective on biochemical adaptation to abiotic stressors in the sea. Ann Rev Mar Sci. (2022) 14:1–23. 10.1146/annurev-marine-022521-102228 34102065

[21] Gelman A Gilks WR Roberts GO. Weak convergence and optimal scaling of random walk metropolis algorithms. Ann Appl Probab. (1997) 7:110–20. 10.1214/aoap/1034625254

[22] Georgiou M Fysikopoulos E Mikropoulos K Fragogeorgi E Loudos G. Characterization of “γ-eye”: a low-cost benchtop mouse-sized gamma camera for dynamic and static imaging studies. Mol Imaging Biol. (2017) 19:398–407. 10.1007/s11307-016-1011-4 27730469

